# Multiple accessory pathways coexisting with a persistent left superior vena cava: a case report

**DOI:** 10.1186/s13256-023-03865-6

**Published:** 2023-03-27

**Authors:** Tetsuya Uemura, Hidekazu Kondo, Tetsuji Shinohara, Masaki Takahashi, Koshiro Akamine, Naoko Ogawa, Kei Hirota, Akira Fukui, Hidefumi Akioka, Kunio Yufu, Naohiko Takahashi

**Affiliations:** Department of Cardiology and Clinical Examination, Faculty of Medicine, Oita University, 1-1 Idaigaoka, Hasama, Yufu, Oita 879-5593 Japan

**Keywords:** Multiple accessory pathways, Persistent left superior vena cava, Wolff–Parkinson–White syndrome, Catheter ablation

## Abstract

**Background:**

Wolff–Parkinson–White syndrome is characterized by a short PR interval (delta-wave), long QRS complex, and the appearance of paroxysmal supraventricular tachycardia. Patients with Wolff–Parkinson–White syndrome usually have one accessory pathway, whereas cases with multiple accessory pathways are rare. Persistent left superior vena cava is a vascular anomaly in which the vein drains into the right atrium through the coronary sinus at the junction of the left internal jugular and subclavian veins due to abnormal development of the left cardinal vein. The simultaneous presence of multiple accessory pathways and persistent left superior vena cava has not been reported before.

**Case presentation:**

A 56-year-old Japanese man with a 5-year history of palpitations was referred for radiofrequency catheter ablation due to increased frequency of tachycardia episodes in the previous 2 months. Persistent left superior vena cava was confirmed by transthoracic echocardiography and computed tomography. An electrophysiological study revealed that the accessory pathways were located in the left lateral wall, anterolateral wall, and posteroseptal region. They were completely ablated with radiofrequency energy application.

**Conclusions:**

We reported an extremely rare case of a patient with multiple accessory pathways and persistent left superior vena cava. Our case may suggest a potential embryological relationship between the multiple accessory pathways and persistent left superior vena cava.

## Background

Wolff–Parkinson–White (WPW) syndrome is characterized by a short PR interval, wide QRS complex, and the appearance of paroxysmal supraventricular tachycardia [1]. The mechanism underlying this syndrome is an accessory atrioventricular connection. Accessory pathways are mostly located around the tricuspid or mitral annulus, accounting for 10–20% and 50–60% of cases of WPW syndrome, respectively [2]. Patients with WPW syndrome usually have one accessory pathway, whereas cases with multiple accessory pathways are rare. Multiple accessory pathways are defined as the presence of two or more pathways separated by at least 1–3 cm. The incidence of multiple accessory pathways was reported to range from 3–20% in surgical studies and from 5–18% in radiofrequency ablation studies [3]. WPW syndrome can be worsened by any infection and chronic medical problem [4–6].

Persistent left superior vena cava (PLSVC) is a vascular anomaly in which the vein drains into the right atrium through the coronary sinus at the junction of the left internal jugular and subclavian veins due to abnormal development of the left cardinal vein. It is the most common vascular anomaly, affecting 0.5% of the general population [7]. Accessory pathways and PLSVC develop at the same embryological stage [8, 9].

We report the case of a 56-year-old man with three accessory pathways and PLSVC. To the best of our knowledge, the simultaneous presence of multiple accessory pathways and PLSVC has not been reported before.

## Case presentation

**Fig. 1:**
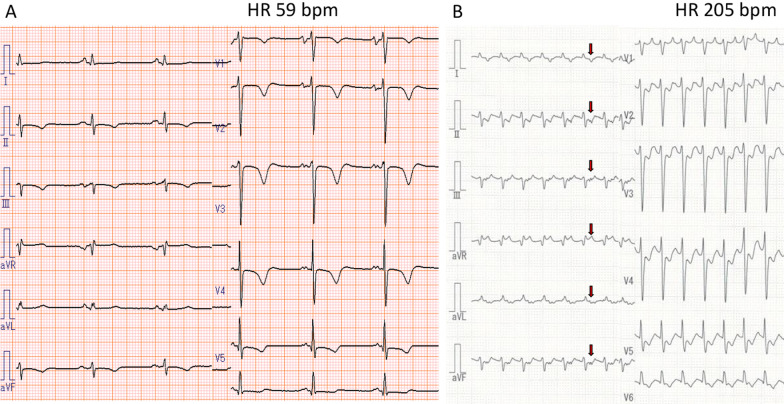
Twelve-lead electrocardiogram at sinus rhythm (**A**) and supraventricular tachycardia (**B**). Red arrows indicate P wave

**Fig. 2:**
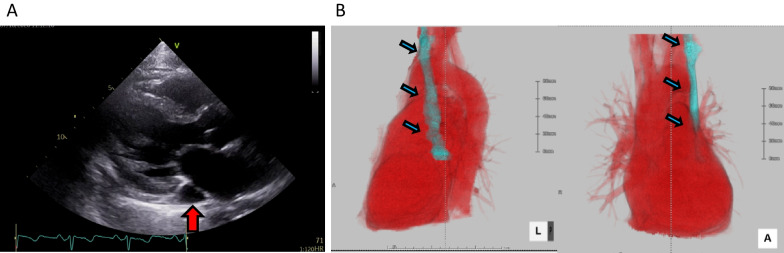
**A** Coronary sinus dilatation on transthoracic echocardiography. **B** Contrast-enhanced computed tomography: the vein draining into the right atrium through the coronary sinus at the junction of the left internal jugular and subclavian veins. Red arrow indicates enlarged coronary sinus, Blue arrows indicates persistent left superior vena cava

A 56-year-old Japanese man with a 5-year history of palpitations was referred for radiofrequency catheter ablation due to increased frequency of tachycardia episodes in the previous 2 months. He had no remarkable physical examination findings, and no remarkable medical, family, and psychosocial history. A 12-lead electrocardiogram during sinus rhythm showed no delta waves (Fig. 1A), whereas an electrocardiogram during palpitations demonstrated regular narrow QRS tachycardia at 200 beats/minute with a negative retrograde P wave in the inferior leads (Fig. 1B). As coronary sinus (CS) dilatation was observed on transthoracic echocardiography (Fig. 2A), we suspected that he had PLSVC. Computed tomography (CT) confirmed that that was correct (Fig. 2B).

After obtaining informed consent from the patient, an electrophysiological study was performed with the patient under sedation with fentanyl and propofol. Two catheters were introduced from the right femoral vein and placed in the right atrium (RA) and right ventricle. An HIS catheter (Biosense Webster, Inc., Irvine, CA, USA) was placed near the His bundle region. A CS-RA catheter was introduced from the right internal jugular vein and advanced to the CS.

**Fig. 3:**
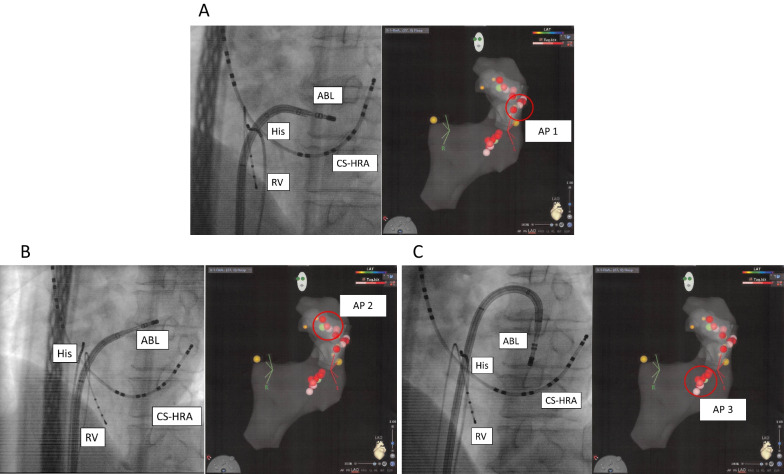
Radiofrequency ablation-delivered points to accessory pathways (APs) located in the lateral (**A**), anterolateral (**B**), and posterolateral (**C**) wall (transparent image and 3D mapping). Red circles indicate the ablation points of accessory pathway 1, 2 and 3

Ventriculoatrial conduction occurred during ventricular pacing, and atrial pre-excitation was observed at CS5-6. During burst pacing from the right ventricle, paroxysmal supraventricular tachycardia was induced. Ventriculoatrial transmits showed the same sequence. This suggested the location of the accessory pathway in the left lateral wall. Mapping of the mitral valve annulus was performed during ventricular pacing using an ablation catheter. The earliest activation site was in the left lateral wall. Radiofrequency energy delivered to this site eliminated the bypass tract (Fig. 3A), and the new earliest activated point was in the anterolateral region of the mitral annulus. The ablation catheter was placed at this site, and it eliminated conduction in the second bypass tract (Fig. 3B). The ventriculoatrial conduction continued, and the third earliest activated point was found in the posteroseptal region of the mitral annulus. The ablation catheter was also placed at this site, and it eliminated conduction in the third bypass tract (Fig. 3C). Persistent ventriculoatrial dissociation was observed, indicating elimination of all accessory pathways. During 12 months of follow-up, he has had no symptoms of palpitation without any drug treatments.

## Discussion

The PLSVC anomaly was found incidentally on computed tomography during the preoperative examination. Moreover, the accessory pathways were identified in the left lateral wall, anterolateral wall, and posteroseptal region. Ablation with the transseptal approach was successful at all three sites.

PLSVC is the remnant of the left cardinal vein, which is formed during the early developmental period. This is the most common vein anomaly, occurring in 0.5% of the general population [7]. Although PLSVC is asymptomatic, it may affect left-sided ablation procedures. Chiang *et al*. reported that CS abnormalities were more common in patients with WPW syndrome than in those with atrioventricular node reentry tachycardia [10]. In their patients, the accessory pathway was located only at the left free wall or in the posteroseptal region. The authors also suggested an anatomical relationship between the distribution of the accessory pathways and major CS abnormalities. During heart formation, the CS arises from the proximal left sinus horn of the sinus venosus at weeks 7–8 of embryonic development. Accessory pathways are considered an extra piece of heart muscle tissue that connects the atrium and the ventricle. This abnormal tissue develops at the same embryonic stage as the CS [11, 12]. Therefore, it seems reasonable to assume that PLSVC and accessory pathways are embryologically related to each other. However, Hwang *et al*. reported that the proportion of PLSVC in patients with supraventricular tachyarrhythmia was 0.27% (18 out of 6662) [13], which is similar to the incidence rate seen in the general population.

## Conclusion

We described a case of a patient with multiple accessory pathways and PLSVC for the first time. The possibility of multiple accessory pathways should thus be considered in patients with PLSVC.

## Data Availability

Not applicable.
